# Branding in children: a barbaric practice still exists in India

**DOI:** 10.11604/pamj.2016.23.62.7968

**Published:** 2016-02-29

**Authors:** Pratap Kumar Patra

**Affiliations:** 1 All India Institute of Medical Sciences, Patna, India

**Keywords:** Branding, harmful, superstition

## Abstract

Branding is an inhuman traditional practice most commonly employed to treat various disorders in neonates and children in certain community in India. Though stringent law exists to prevent such harmful practices, cases of branding is not uncommon in current era.

## Introduction

Branding is a process by which a mark or symbol is burned into the skin of a living person. It is done by using a hot iron rod. In ancient era, it was being used as punishment or identifies an enslaved or oppressed person. Branding in India has remained mostly as a traditional practice for various disease conditions in children. Though the incidence of branding is now rare in developed nation, this practice is yet to be abolished from our country. We recently came across with a child of Wilson's disease with multiple circumscribed scarring lesions over the abdomen because of this superstitious practice.

## Patient and observation

**Figure 1:**
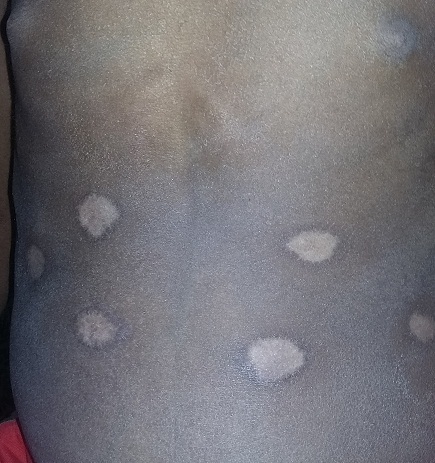
Hypopigmented lesions over the abdomen following branding

A 10 year male child was brought to Pediatric emergency with history of jaundice for 6 months, swelling of body for forty five days and altered sensorium for seven days. On general physical examination he had moderate pallor, severe jaundice and generalised body edema. He also noticed to have six hypopigmented circumscribed macular lesion over the abdomen (Figure 1). On further inquiry parents came out with history of branding in native place which also followed by pus discharge from these lesions and required further treatment. On systemic examination he had abdominal distension, hepatomegaly and there was evidence of free fluid in the abdomen. His Glasgow coma score was 8 and central nervous system examination revealed exaggeration of deep tendon reflex and plantar reflex were up going. He was transferred to Pediatric intensive care unit and managed successfully for grade II hepatic encephalopathy. His ophthalmological examination revealed Kayser-Fleisher ring (copper deposits in the outer rim of the cornea) and serum ceruloplasmin value was corroborated with the diagnosis of Wilsons disease. He was started on d-penicillamine and successfully discharged after eighteen day of hospitalisation.

## Discussion

The etymology of the word "brand" is traced back to 12^th^ century Norse brena which meant to burn to light [1]. Between 16th to 18th century it was being used as punishment measures across America, Europe, Rome and Britain especially for slaves. In our country, there is history of practice of branding as a religious tradition by certain section of the community from south India. There were reports of neonatal branding in large scale in 1991 in past from the rural part of India [2]. It has been used as a treatment measures for convulsion, seizure disorder, Hepatosplenomegaly and jaundice in children [3]. Death has also been reported following this procedure in newborns [2]. In our case though, the child developed local septicemia at the site of branding, he recovered following treatment. The root cause of the persistence of this draconian practice is illiteracy, superstition and lack of access to standard health care. It is a criminal offense under Indian Penal code 324 and paediatrician should be aware that this can be a form of severe child abuse. This inhumane practice should be condemned by all society. The perpetrators who foster this practice should be punished severely. It is also essential to provide mass education to the community where this harmful practice is prevailing most, then only can it be abolished.

## Conclusion

To conclude, branding is a superstitious practice still prevailing in 21^st^ century. Providing standard health care to people at their doorstep and educating them about the harm of this practice can reduce such diabolical practice.
